# A Novel Analytical Model for the IEEE 802.11p/bd Medium Access Control, with Consideration of the Capture Effect in the Internet of Vehicles

**DOI:** 10.3390/s23239589

**Published:** 2023-12-03

**Authors:** Yang Wang, Jianghong Shi, Zhiyuan Fang, Lingyu Chen

**Affiliations:** 1 School of Informatics, Xiamen University, Xiamen 361005, China; yangwang@stu.xmu.edu.cn (Y.W.); chenly@xmu.edu.cn (L.C.); 2 China Mobile Communications Group Shanxi Co., Ltd., Taiyuan 030032, China; fangzhiyuan@sx.chinamobile.com

**Keywords:** vehicular ad hoc network, internet of vehicles, IEEE 802.11p/bd, medium access control, capture effect, Nakagami-*m* fading

## Abstract

The traditional vehicular ad hoc network (VANET), which is evolving into the internet of vehicles (IoV), has drawn great attention for its enormous potential in road safety improvement, traffic management, infotainment service support, and even autonomous driving. IEEE 802.11p, as the vital standard for wireless access in vehicular environments, has been released for more than one decade and its evolution, IEEE 802.11bd, has also been released for a few months. Since the analytical models for the IEEE 802.11p/bd medium access control (MAC) play important roles in terms of performance evaluation and MAC protocol optimization, a lot of analytical models have been proposed. However, the existing analytical models are still not accurate as a result of ignoring some important factors of the MAC itself and real communication scenarios. Motivated by this, a novel analytical model is proposed, based on a novel two-dimensional (2-D) Markov chain model. In contrast to the existing studies, all the important factors are considered in this proposed model, such as the backoff freezing mechanism, retry limit, post-backoff states, differentiated packet arrival probabilities for empty buffer queue, and queue model of packets in the buffer. In addition, the influence of the capture effect under a Nakagami-*m* fading channel has also been considered. Then, the expressions of successful transmission, collided transmission, normalized unsaturated throughput, and average packet delay are all meticulously derived, respectively. At last, the accuracy of the proposed analytical model is verified via the simulation results, which show that it is more accurate than the existing analytical models.

## 1. Introduction

**Figure 1:**
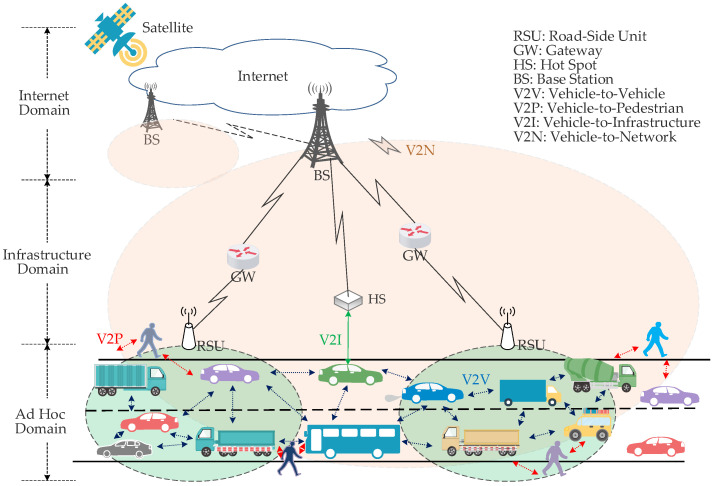
Heterogeneous vehicular communications structure.

The vehicular ad hoc network (VANET) has been a widespread concern of academia and industry for its enormous potential in improving road safety, promoting traffic efficiency, providing infotainment services, and even supporting autonomous driving [1,2]. Benefiting from the rapid development of information and communication technology [3,4,5,6,7,8,9,10,11], the traditional VANET is evolving into the internet of vehicles (IoV) [12]. It can support heterogeneous vehicular communication modes, including vehicle-to-vehicle (V2V), vehicle-to-pedestrian (V2P), vehicle-to-infrastructure (V2I) and vehicle-to-network (V2N), as shown in Figure 1, for satisfying the requirements of different safety or non-safety applications [13,14]. However, the key to accomplish differentiated applications depends on whether the vehicles effectively access the wireless channel. As an important channel access standard for IoV, IEEE 802.11p outlines the specifications of the physical (PHY) layer and medium access control (MAC) layer, where the latter includes the distributed coordination function (DCF) and enhanced distributed channel access (EDCA). According to the EDCA, four access categories (ACs) are defined. In fact, each AC queue is called an enhanced distributed channel access function (EDCAF), which is an enhanced variant of the DCF. It contends for the transmission opportunity (TXOP) by using a set of EDCA parameters [15]. Actually, IEEE 802.11bd, as an evolving version of IEEE 802.11p, also adopts DCF and EDCA protocols [16]. Due to the fact that the DCF protocol is the essential channel access protocol of IEEE 802.11p/bd, it is necessary to propose an effective analytical model for evaluating the precise performance of IEEE 802.11p/bd DCF (or EDCA) in IoV.

Since the DCF is the basis of the IEEE 802.11 series of standards, a lot of analytical models based on Bianchi’s pioneering work in [17] have been proposed, under different assumptions, in different communication scenarios [15,18,19,20,21,22,23,24,25,26,27,28,29,30,31,32,33,34,35,36,37,38,39,40,41,42,43]. However, none of them can completely show all the characteristics of the DCF and analyze the performance of the DCF precisely in IoV scenarios, especially for those ignoring the capture effect [17,18,19,20,21,22,23,24,25,26,27,28,29,30,31,32,33,34,35]. In fact, when the receiver receives the signal power from one transmitter that is higher than that of the other transmitters, the capture effect, which is a common phenomenon in wireless communication, may occur [43,44,45]. According to [45], the system performance of IEEE 802.11 networks can be improved by the capture effect. Nevertheless, there are only a few analytical models in the existing literature considering the capture effect under frequently used fading channels in vehicular communication [36,37,38,39,40,41,42,43]. According to [46,47], the Nakagami-*m* channel model represents small-scale fading in vehicular communication and reflects a realistic driving environment. Therefore, analyzing IEEE 802.11p/bd DCF under this fading channel model is necessary to show its real performance in IoV. Though the capture effect under the Nakagami-*m* fading channel is considered in [42], the authors only show the non-closed-form formulation for the normalized throughput. In addition, the capture effect under the Nakagami-*m* fading channel is also considered in our previous work in [43] under the saturated condition, which is a special case that all vehicles in the network always have packets to be transmitted. In fact, the vehicles are often under an unsaturated condition, which means that the buffer queues of vehicles do not always have packets waiting to be transmitted [26,48,49]. Motivated by this, we make the performance analysis of IEEE 802.11p/bd DCF more accurate by proposing a novel two-dimensional (2-D) Markov chain model, where all important characteristics of the DCF itself are included, and the capture effect under the Nakagami-*m* fading channel is considered, to make the analytical procedure more reasonable. To the best of our knowledge, it is the first analysis of the unsaturated performance of IEEE 802.11p/bd DCF with consideration of the capture effect under the Nakagami-*m* fading channel by proposing a novel 2-D Markov model different from the existing ones. The contributions of this paper are threefold.

1. A novel 2-D Markov chain model is proposed, which is different from the existing ones. In the proposed 2-D Markov chain model, all the key characteristics of the DCF are considered, i.e., backoff freezing mechanism, immediate access mechanism, finite retry limit, post-backoff procedure, different packet arrival probabilities under different channel states for the empty buffer and queuing model of the buffer queue.
2. The capture effect under a Nakagami-*m* fading channel is considered. Then, the closed-form expressions of successful transmission, collided transmission, normalized unsaturated throughput, and average packet delay are all meticulously derived, respectively.
3. To verify the accuracy of the proposed model, simulation results are given. In addition, it is also compared with the existing analytical models. As expected, the proposed model is more accurate than the existing models in terms of normalized unsaturated throughput and average packet delay.

The rest of this paper is organized as follows. Section 2 surveys the related research. Section 3 presents an overview of the DCF and develops a novel analytical model for the DCF. Section 4 validates the accuracy of the proposed analytical model for the DCF by comparing it with the existing models. Finally, Section 5 concludes the paper. In addition, Table 1 presents a list of abbreviations used in the paper.

## 2. Related Work

Due to the wide usage of the IEEE 802.11 series of standards in wireless local area networks, a lot of analytical models for the adopted MAC protocols (i.e., DCF or EDCA) under different network scenarios have been proposed for evaluating its performance and then designing a MAC protocol meeting the requirements of different scenarios by researchers around the world.

As is known, Bianchi, G. first proposed a 2-D Markov chain model (which is called Bianchi’s model [17]) to analyze the DCF protocol under ideal channel conditions and saturated conditions, without considering the backoff freezing mechanism and retry limit. Based on this pioneering work, a lot of research work has been conducted by worldwide researchers. For example, Duffy, K. et al. extended Bianchi’s model to the nonsaturated condition [18], while Madhavi, T. et al. modeled collision-alleviating DCF with a finite retry limit [19]. Though the backoff freezing mechanism and unsaturated condition are considered in [20], the finite retry limit and post-backoff procedure are missed. In addition, the finite retry limit is included in the model in [21], but the post-backoff procedure is still missed. In [22], the performances of saturated throughput and delay for the DCF based on [17], with consideration of the finite retry limit and backoff freezing mechanism, are analyzed. In addition, IEEE 802.11p DCF is analyzed and optimized under saturation conditions in [23], which is just based on the 2-D Markov chain model in [22]. Though the performances of saturated throughput and delay for IEEE 802.11p EDCA are analyzed in [24], the difference between the 2-D Markov chain models in [17,24] is the consideration of retry limit. Moreover, in [15,25], IEEE 802.11p EDCA is analyzed under unsaturation conditions with consideration of the retry limit and backoff freezing mechanism, while the latter considers the queuing model and ignores the post-backoff procedure. Moreover, Cao et al. analyzed the EDCA with consideration of the backoff freezing mechanism, finite retry limit and idle state for four ACs [26], which is more accurate than the model proposed in [15] for considering the queuing model. However, these studies are all on the basis of ideal channel conditions, which is not in line with the reality that the channel does have an effect on the DCF or EDCA protocol in IoV.

Hence, Zhang, Y. et al. analyzed the DCF based on [17] under different channel conditions [27]. In addition, Peng, H. et al. presented a probabilistic analysis of the DCF in a multiplatooning scenario, while a constant probability is used for the transmission error of a packet [28]. However, the backoff freezing mechanism and post-backoff procedure are both ignored. Therefore, Almohammedi, A.A. et al. considered the backoff freezing mechanism and unsaturated condition in the 2-D Markov chain model and analyzed the throughput of the DCF under a varying bit-error ratio (BER) [29]. In addition, Peng, J. et al. also investigated the impact of channel transmission error with a constant probability for a packet in [30]. Moreover, Alshanyour, A. et al. evaluated IEEE 802.11 DCF based on a three-dimensional (3-D) Markov chain model under saturated conditions, and just a constant BER was considered [31]. Meanwhile in [32], an hierarchical 3-D Markov model was proposed for analyzing the non-saturated IEEE 802.11 DCF-based networks under error-prone channel conditions (accomplished by varying the constant block error probability). In [33], Wang, N. et al. evaluated the IEEE 802.11p EDCA based on a 3-D Markov chain model under both saturated and unsaturated conditions, while the impact of channel fading and modulation was modeled with a constant BER for simplicity. In addition, Harkat, Y. et al. analyzed the saturation throughput and average access delay with a constant BER too [34]. In [35], a 3-D Markov chain model is also used to analyze the throughput and average access delay for EDCA under different values of BER.

Unfortunately, the above-mentioned analytical models all ignore the influence from the capture effect. Since the capture effect exists in wireless communication systems, it is necessary to consider it to make the analytical results more accurate [36,44,45,50]. Therefore, Shah, A.F.M.S. et al. analyzed the saturation throughput of DCF by considering the capture effect in a Rayleigh fading environment based on Bianchi’s model [36]. In [37], Lei, L. et al. analyzed the saturation throughput of the DCF with the consideration of the capture effect under the free-space propagation model based on a 3-D Markov chain model. However, the backoff freezing mechanism and post-backoff procedure are both disregarded. Meanwhile in [38,39], Daneshgaran, F. et al. analyzed the saturation throughput and unsaturated throughput with consideration of the capture effect under the Rayleigh fading channel, respectively. However, they ignored the post-backoff procedure and retry limit in their 2-D Markov models. In [40], Han, H. et al. also gave the saturation throughput of the DCF with consideration of the capture effect in a Rayleigh fading channel and the retry limit. Again, in [41], Sutton G.J. et al. modeled the DCF with the capture effect under a Rayleigh fading channel based on a 3-D Markov model, but the backoff freezing mechanism was ignored. Moreover, Leonardo, E. J. et al. analyzed throughput of the DCF with consideration of the capture effect under Hoyt, Rice, and Nakagami-*m* fading channels in [42], while ignoring the backoff freezing mechanism and the retry limit. In addition, the closed-form expressions of capture effect and throughput are missed. Though the capture effect under the Nakagami-*m* fading channel is included in our previous work [43], the saturation condition is assumed for convenience, which makes this model not very in line with reality.

Therefore, a novel analytical model considering all the important factors (i.e., backoff freezing mechanism, immediate access mechanism, finite retry limit, post-backoff procedure, different packet arrival probabilities under different channel states for the empty buffer, queuing model of buffer queue, and the capture effect under a Nakagami-*m* fading channel) is proposed for the performance analysis of IEEE 802.11p/bd DCF in real vehicular communication scenarios. Then, we carefully derive the closed-form expressions of successful transmission, collided transmission, unsaturated throughput and average packet delay, respectively. In fact, the proposed analytical model can be easily extended to the performance analysis of the EDCA. Similar extension methods can be referenced in [15,25,26].

## 3. The Proposed Analytical Model

In this section, a novel analytical model is proposed to evaluate the performance of the IEEE 802.11p/bd DCF protocol. Different from the existing work, we develop a novel 2-D Markov model to derive the closed-form expressions of normalized unsaturated throughput and average packet delay, which are the two main commonly used evaluation indicators. For convenience, the significant notations and variables used in the analysis procedure are summarized in Table 2.

### 3.1. Brief Description of DCF

According to the DCF protocol, the vehicles in the network contend for the wireless channel by the carrier sense multiple access with collision avoidance (CSMA/CA) mechanism, which is based on the slotted binary exponential backoff (BEB) scheme. In fact, each vehicle with a packet to be transmitted needs to sense the channel before transmission. If the channel is idle for a duration exceeding the distributed interframe space (DIFS), the vehicle transmits the packet. This is a so-called immediate access mechanism. Otherwise, the backoff procedure is invoked to defer the transmission to avoid collision. According to the BEB scheme, the random backoff time is uniformly chosen in the range [0,CW−1], where CW is the contention window with the minimum ${CW}_{min}=W_{0}$. The backoff counter is decremented by one at the end of each idle slot and the vehicle transmits immediately when the backoff counter reaches zero. However, if the channel is busy, the backoff counter will be frozen. When the channel is idle again for more than one DIFS, the backoff counter will be resumed. The transmission for a data packet (DATA) from the source vehicle is successful if an acknowledgement (ACK) from the destination vehicle can be received by the source vehicle after a period of short interframe space (SIFS). Otherwise, this transmission has failed and a retransmission is scheduled by starting another backoff period with CW doubled. If the maximum of contention window (${{CW}_{max}=W}_{M}=2^{M}W_{0}$) is reached and then CW can be set to $W_{M}$ at most for f times before discarding this packet. Hence, the value of CW is reset to ${CW}_{min}$ after a successful transmission or being discarded due to reaching the retry limit (M+f). A backoff procedure shall be performed immediately after the end of every transmission, even if no additional transmissions are currently queued. This is the so-called post-backoff mechanism.

The basic access mode and the request-to-send/clear-to-send (RTS/CTS) access mode are two access techniques supported by the DCF protocol. In fact, the basic access mode is a two-way handshaking mechanism using DATA/ACK packets, while the RTS/CTS access mode is a four-way handshaking mechanism using RTS/CTS packets to reserve the channel resource before transmission. In fact, the latter follows the same backoff rules as the former and reduces the risk of large packet collision by short RTS/CTS packets. Since the duration of the ongoing transmission is included in the above-mentioned control packet, each vehicle updates its network allocation vector (NAV) by the RTS or CTS and then defers transmission for a specified duration to avoid collision.

### 3.2. A Novel 2-D Markov Chain Model

**Figure 2:**
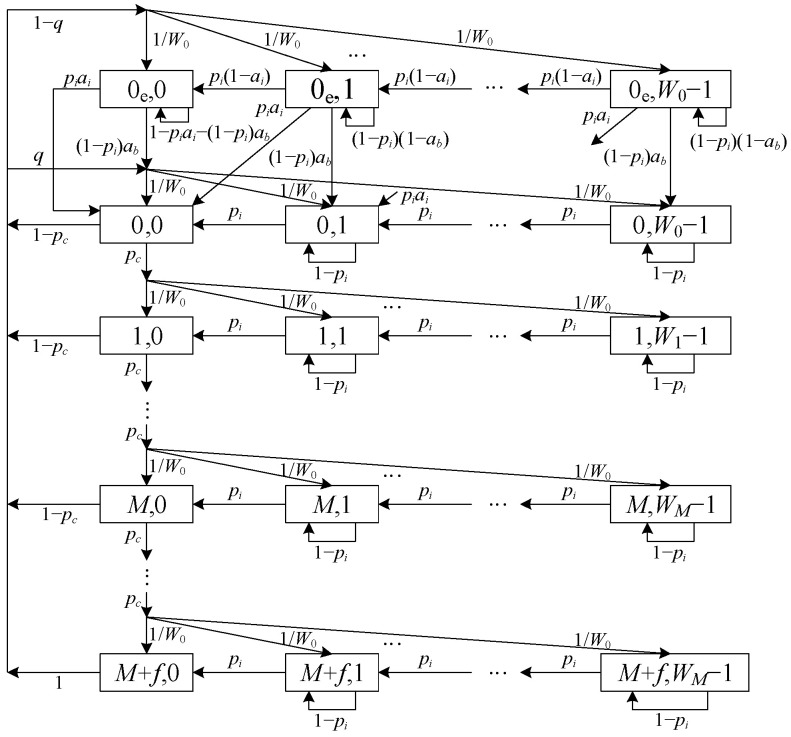
The proposed 2-D Markov chain model.

In Figure 2, a novel 2-D Markov chain model is proposed for modeling the behavior of the DCF protocol in IoV. In this Markov chain model, the unsaturated condition is considered, i.e., the buffer of each vehicle will be empty with probability (1−q), where q denotes the probability that there exist packets in the buffer after a successful transmission or dropping a packet due to reaching the retry limit. The states for vehicles at time t are represented as $\left\{s\left(t\right),b(t)\right\}$, where $s\left(t\right)$ with values from {0, 1,…, M+f} is defined as the random backoff stage and b(t) with values from {0, 1,…, $W_{i}-1$}) is defined as the value of the backoff counter at time t. Moreover, the states $\left\{{s\left(t\right)}_{e},b(t)\right\}$ refer to the states with empty buffer, which means that the buffer queue of one vehicle is empty after a successful transmission or a failure. These random variables are dependent because the maximum value of the backoff counter depends on the backoff stage

(1) $$W_{i}=\left\{\begin{array}{c} \begin{array}{c} \begin{array}{cc} 2^{i}W_{0}, & 0\leq i\leq M \end{array} \end{array}\\ \begin{array}{cc} W_{M}, & M<i\leq M+f \end{array} \end{array}\right.$$

Let b(j,k) be the stationary distribution of the 2-D Markov chain model in Figure 2. Then, the one-step state transition probabilities can be expressed as

(2) $$\left\{\begin{array}{c} P\left\{(0_{e},k-1)\left|\left(0_{e},k\right)\right.\right\}=p_{i}(1-a_{i}),\;1\leq k\leq W_{0}-1\\ P\left\{(0,k-1)\left|\left(0_{e},k\right)\right.\right\}=p_{i}a_{i},\;1\leq k\leq W_{0}-1\\ P\left\{(0,k)\left|\left(0_{e},k\right)\right.\right\}=(1-p_{i})a_{b},\;1\leq k\leq W_{0}-1\\ P\left\{(0_{e},k)\left|\left(0_{e},k\right)\right.\right\}=(1-p_{i})(1-a_{b}),\;1\leq k\leq W_{0}-1\\ P\left\{(0,k)\left|\left(0_{e},0\right)\right.\right\}=(1-p_{i})a_{b}/W_{0},\;1\leq k\leq W_{0}-1\\ P\left\{(0_{e},0)\left|\left(0_{e},0\right)\right.\right\}=1-p_{i}a_{i}-(1-p_{i})a_{b}\\ P\left\{(0,0)\left|\left(0_{e},0\right)\right.\right\}=p_{i}a_{i}+(1-p_{i})a_{b}/W_{0}\\ P\left\{(j,k-1)\left|\left(j,k\right)\right.\right\}=p_{i},\;0\leq j\leq M+f,1\leq k\leq W_{j}-1\\ P\left\{(j,k)\left|\left(j,k\right)\right.\right\}=1-p_{i},\;0\leq j\leq M+f,1\leq k\leq W_{0}-1\\ P\left\{(0,k)\left|\left(j,0\right)\right.\right\}=(1-p_{c})q/W_{0},\;0\leq j<M+f,0\leq k\leq W_{0}-1\\ P\left\{(0_{e},k)\left|\left(j,0\right)\right.\right\}=\left(1-p_{c})(1-q\right)/W_{0},\;0\leq j<M+f,0\leq k\leq W_{0}-1\\ P\left\{(j+1,k)\left|\left(j,0\right)\right.\right\}=p_{c}/W_{\min(j+1,M)},\;0\leq j<M+f,0\leq k\leq W_{0}-1\\ P\left\{(0,k)\left|\left(M+f,0\right)\right.\right\}=q/W_{0},\;0\leq k\leq W_{0}-1\\ P\left\{(0_{e},k)\left|\left(M+f,0\right)\right.\right\}=\left(1-q\right)/W_{0},\;0\leq k\leq W_{0}-1 \end{array}\right.$$

Therefore, based on Figure 2 and Equation (2), we can further obtain the following steady-state probabilities, i.e.,

(3) $$b(j,0)={\left(p_{c}\right)}^{j}b(0,0),\;1\leq j\leq M+f$$

(4) $$b(j,k)=\frac{(W_{j}-k){\left(p_{c}\right)}^{j}}{W_{j}p_{i}}b(0,0)$$

(5) $$b(0,0)=(1-p_{c})\sum_{j=0}^{M+f-1}b(j,0)+b(M+f,0)$$

(6) $$b(0_{e},0)=b(0,0)\frac{1-q}{W_{0}(a_{b}+p_{i}a_{i}-p_{i}a_{b})}\left(1+\sum_{k=1}^{W_{0}-1}{\left(\frac{p_{i}-p_{i}a_{i}}{a_{b}+p_{i}-p_{i}a_{b}}\right)}^{k}\right)$$

The detail derivation processes of the above expressions are omitted to save space, and interested readers are encouraged to refer to [15,17]. By using the above expressions, we can easily obtain

(7) $$\sum_{j=1}^{M+f}\sum_{k=0}^{W_{j}-1}b(j,k)=b(0,0)\left\{\frac{p_{c}-{\left(p_{c}\right)}^{M+f+1}}{1-p_{c}}\right.+\frac{1}{2p_{i}}\left[\frac{2W_{0}p_{c}(1-{\left(2p_{c}\right)}^{M})}{1-2p_{c}}\right.\left.\left.+\frac{W_{0}2^{M}{\left(p_{c}\right)}^{M+1}(1-{\left(p_{c}\right)}^{f})+{\left(p_{c}\right)}^{M+f+1}-p_{c}}{1-p_{c}}\right]\right\}$$

(8) $$\sum_{k=0}^{W_{0}-1}b(0_{e},k)+\sum_{k=0}^{W_{0}-1}b(0,k)=\left[1+\frac{W_{0}-1}{2p_{i}}\right]b(0,0)+\left[1+\frac{(W_{0}-1)(1-p_{i})a_{b}}{2p_{i}}\right]b(0_{e},0)$$

Then, according to the normalization condition for stationary distribution, we have

(9) $$\sum_{k=0}^{W_{0}-1}b(0_{e},k)+\sum_{j=0}^{M+f}\sum_{k=0}^{W_{j}-1}b(j,k)=1$$

After substituting Equations (6)–(8) into Equation (9), we can obtain

(10) $$\begin{array}{ccc} \frac{1}{b(0,0)} & = & 1+\frac{W_{0}-1}{2p_{i}}+\frac{p_{c}-{\left(p_{c}\right)}^{M+f+1}}{1-p_{c}}+\frac{1-q}{W_{0}(a_{b}+p_{i}a_{i}-p_{i}a_{b})}\cdot \left[1+\frac{(W_{0}-1)(1-p_{i})a_{b}}{2p_{i}}\right]\left[1+\sum_{k=1}^{W_{0}-1}{\left(\frac{p_{i}-p_{i}a_{i}}{a_{b}+p_{i}-p_{i}a_{b}}\right)}^{k}\right]\\ & & +\frac{1}{2p_{i}}\left[\frac{2W_{0}p_{c}(1-{\left(2p_{c}\right)}^{M})}{1-2p_{c}}\right.\left.+\frac{W_{0}2^{M}{\left(p_{c}\right)}^{M+1}(1-{\left(p_{c}\right)}^{f})+{\left(p_{c}\right)}^{M+f+1}-p_{c}}{1-p_{c}}\right] \end{array}$$

Therefore, the probability that a concerned vehicle transmits in a randomly chosen slot can be expressed as

(11) $$\tau_{tra}=\sum_{j=0}^{M+f}b(j,0)=\frac{1-{\left(p_{c}\right)}^{M+f+1}}{1-p_{c}}b(0,0)$$

Then, substituting (10) into (11), we can obtain

(12) $$\begin{array}{ccc} \tau_{tra} & = & \frac{1-{\left(p_{c}\right)}^{M+f+1}}{1-p_{c}}\left\{1+\frac{W_{0}-1}{2p_{i}}+\frac{p_{c}-{\left(p_{c}\right)}^{M+f+1}}{1-p_{c}}\right.+\frac{1-q}{W_{0}(a_{b}+p_{i}a_{i}-p_{i}a_{b})}\left[1+\frac{(W_{0}-1)(1-p_{i})a_{b}}{2p_{i}}\right]\\ & & \cdot \left[1+\sum_{k=1}^{W_{0}-1}{\left(\frac{p_{i}-p_{i}a_{i}}{a_{b}+p_{i}-p_{i}a_{b}}\right)}^{k}\right]+\frac{1}{2p_{i}}\left[\frac{2W_{0}p_{c}(1-{\left(2p_{c}\right)}^{M})}{1-2p_{c}}\right.{\left.\left.+\frac{W_{0}2^{M}{\left(p_{c}\right)}^{M+1}(1-{\left(p_{c}\right)}^{f})+{\left(p_{c}\right)}^{M+f+1}-p_{c}}{1-p_{c}}\right]\right\}}^{-1} \end{array}$$

where $a_{i}$ and $a_{b}$ are the probabilities of packet arrivals during an idle slot and a busy slot, respectively. If the arrival of a packet obeys Possion distribution, these two values are calculated as

(13) $$\left\{\begin{array}{c} a_{i}=\sum_{k=1}^{\infty }\frac{{\left(\lambda \sigma \right)}^{k}}{k!}e^{-\lambda \sigma }=1-\frac{{\left(\lambda \sigma \right)}^{0}}{0!}e^{-\lambda \sigma }=1-e^{-\lambda \sigma }\\ a_{b}=\sum_{k=1}^{\infty }\frac{{\left(\lambda T_{b}\right)}^{k}}{k!}e^{-\lambda T_{b}}=1-\frac{{\left(\lambda T_{b}\right)}^{0}}{0!}e^{-\lambda T_{b}}=1-e^{-\lambda T_{b}} \end{array}\right.$$

where σ is the duration of an idle slot and $T_{b}$ is the duration of a busy slot. Here, the durations of a successful slot and a collided slot are assumed to be the same for simplicity. In addition, the probability that the channel is idle for the vehicle concerned is calculated as

(14) $$p_{i}={\left(1-\tau_{tra}\right)}^{n-1}$$

where n is the number of vehicles and $\tau_{tra}$ is the transmission probability calculated by Equation (12). Because of the consideration of the capture effect, the probability of a collided transmission in a given slot can be calculated by

(15) $$p_{c}=\sum_{k=1}^{n-1}\left[1-\frac{p_{cap}(k+1,z_{0})}{k+1}\right]C_{n-1}^{k}{\left(\tau_{tra}\right)}^{k}{\left(1-\tau_{tra}\right)}^{n-k-1}$$

where $C_{n-1}^{k}=(n-1)!/\left[k!\left(n-k-1\right)!\right]$ and $p_{cap}(\cdot ,\cdot )$ is the occurrence probability of capture effect. According to [45,51], the capture effect occurs at the targeted vehicle if the received signal power from some vehicles is larger than the sum of the others’. For an inference-limited system, the capture condition is $\gamma_{t}/\sum_{k=1,k\neq t}^{n}\gamma_{k}>z_{0}$, where $\gamma_{t}$, $\gamma_{k}$ and $z_{0}$ are the signal power from one vehicle, the interference signal power from the other vehicles, and the capture threshold, respectively. Under the hypothesis of perfect power control, the capture probability conditioned on n−1 interferers (n≥2) can be calculated by [51]

(16) $$\begin{array}{cc} p_{cap}(n,z_{0}) & =n\int_{0}^{\infty }f_{\gamma_{t}}(\gamma_{t})\cdot \mathrm{Pr}\left[\gamma_{t}/\sum_{k=1,k\neq t}^{n}\gamma_{k}>z_{0}\right]d\gamma_{t}\\ & =n\int_{0}^{\infty }f_{\gamma_{t}}(\gamma_{t})\left[\int_{0}^{\gamma_{t}/z_{0}}f_{\gamma_{n-1}}(\gamma_{n-1})d\gamma_{n-1}\right]d\gamma_{t} \end{array}$$

where $f_{\gamma_{t}}(\gamma_{t})$ is the instantaneous received power and $f_{\gamma_{n-1}}(\gamma_{n-1})$ is the (n−1)-fold convolution of $f_{\gamma_{t}}(\gamma_{t})$. That the Nakagami-*m* fading is more suitable to the IoV scenario leads to its wide adoption in the research of VANETs [52]. Therefore, the Nakagami-*m* fading channel is considered here. Then, $f_{\gamma_{t}}(\gamma_{t})$ and $f_{\gamma_{n-1}}(\gamma_{n-1})$ of (16) can be given by

(17) $$f_{\gamma_{t}}(\gamma_{t})=\frac{m^{m}\gamma_{t}{}^{m-1}}{{\bar{\gamma }}^{m}\Gamma (m)}e^{-\frac{m\gamma_{t}}{\bar{\gamma }}},\;\gamma \geq 0$$

(18) $$f_{\gamma_{n-1}}(\gamma_{n-1})=\frac{m^{m(n-1)}}{{\bar{\gamma }}_{}^{m(n-1)}\Gamma (mn-m)}\gamma_{n-1}^{m(n-1)-1}e^{-\frac{m\gamma_{n-1}}{\bar{\gamma }}}$$

where m∈[1/2,∞) is the shape parameter. $\bar{\gamma }{=P}_{tx}\cdot C\cdot r_{i}^{-\alpha }$ is the average received power determined by transmission power ($P_{tx}$), path-loss exponent (α), and a constant related to the antenna gains (C). Besides, for all vehicles in the network, the carrier frequency and the speed of light are both the same. According to our previous work in [52], the capture probability (i.e., Equation (16)) can be further expressed as

(19) $$p_{cap}(n,z_{0})=\frac{n}{\Gamma (m)\Gamma (mn-m)}\sum_{k=0}^{\infty }\frac{{\left(-1\right)}^{k}\Gamma (mn+k)}{k!(mn-m+k)z_{0}{}^{mn-m+k}}$$

**Figure 3:**
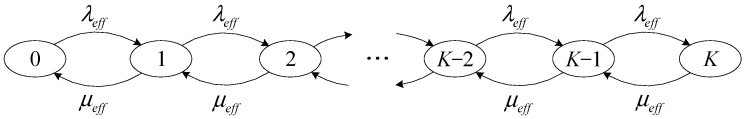
State transition diagram for an *M*/*M*/1/*K* queue.

According to the numerical method in [17], we can obtain $\tau_{tra}$ and $p_{c}$ by figuring out the equation set (the non-linear system) composed of Equations (12) and (15) after submitting (13) into (12) and (19) into (15), respectively. Though the capture threshold ($z_{0}$) and the shape parameter (m) of this equation set are given in advance, the probability that there is at least one packet in the vehicle buffer (q) is still unknown, which is related to the service intensity ρ determined by the arrival rate of packets and the service rate. Here, we treat each vehicle as an *M*/*M*/1/*K* queue with a first-in-first-out (FIFO) policy (as shown in Figure 3), where the packet arrival of each buffer from the upper layer is a Possion process with rate λ (in packets per second, pkts/s) and the interval of service time for each packet is exponentially distributed with mean value $1/\mu_{eff}$. In addition, for each buffer queue, the maximum length is *K* (including the packet in service).

According to [53], the probability that the buffer queue of any vehicle is non-empty is given by

(20) $$q=1-\frac{1-\frac{\lambda_{pkt}}{\mu_{eff}}}{1-{\left(\frac{\lambda_{pkt}}{\mu_{eff}}\right)}^{K+1}}$$

where $\rho =\frac{\lambda_{pkt}}{\mu_{eff}}\neq 1$ and the effective packet service rate is given by

(21) $$\mu_{eff}=\mu_{suc}+\mu_{dis}$$

where $\mu_{suc}$ (i.e., the maximum service rate of packet successful transmission for a concerned vehicle) is given by

(22) $$\mu_{suc}=\frac{\sum_{j=0}^{n-1}\frac{1}{j+1}p_{cap}(j+1,z_{th})C_{n-1}^{j}{\left(\tau_{tra}^{sat}\right)}^{j}{\left(1-\tau_{tra}^{sat}\right)}^{n-j-1}}{\sigma_{ave}^{sat}}$$

where $\tau_{tra}^{sat}$ and $\sigma_{ave}^{sat}$ are the average slot time and the transmission probability at saturation, respectively. Moreover, $p_{cap}(\cdot ,\cdot )$ is the capture probability expressed as (19) with additionally $p_{cap}\left(1,\cdot \right)=1$ (i.e., that only one vehicle transmits leading to a successful transmission). In addition, the rate at which packets are being discarded due to reaching the retry limit can be calculated by

(23) $$\mu_{dis}=\frac{{\left(p_{c}^{sat}\right)}^{M+f+1}}{\sigma_{ave}^{sat}}$$

Since $\tau_{tra}^{sat}$, $p_{c}^{sat}$, and $\sigma_{ave}^{sat}$ are the values of transmission probability, collision probability and the average length of a virtual slot at saturation, we can substitute $\rho =a_{i}=a_{b}=1$ into (12) and numerically solve a non-linear system for their values. The detailed steps for finding these two values can be found in our previous work in [43]. Therefore, after substituting (21), (22) and (23) into (20), the value of q can be obtained by a given value of λ. Finally, the equation set composed of (12) and (15) with unknown parameters $\tau_{tra}$ and $p_{c}$ can be numerically solved with a unique solution.

### 3.3. Calculation of Normalized Throughput

Let η be the normalized throughput. Since it is the ratio of the duration of successful transmission of the packet payload ($T_{L_{p}}$) to the average length of a virtual slot ($\sigma_{ave}$), it can be calculated as

(24) $$\eta =\frac{p_{suc}^{}p_{tra}T_{L_{p}}}{\sigma_{ave}}$$

where $T_{L_{p}}=\frac{L_{p}}{R_{t}}$, $L_{p}$ is the payload of the transmitted packet (which is usually assumed the same for all packets for simplicity) and $R_{t}$ is the data transmission rate. $p_{tra}$ is the probability that one or more of the vehicles transmit in a certain slot, $p_{suc}$ denotes the probability that one vehicle successfully transmits in a certain slot on the conditioned that one or more of the vehicles transmit. Then, the average length of a virtual slot can be calculated as

(25) $$\sigma_{ave}=(1-p_{tra})\sigma +p_{suc}^{}p_{tra}T_{s}+p_{tra}(1-p_{suc}^{})T_{c}$$

where σ, $T_{s}$, $T_{c}$ denote the average durations of an idle slot, successful transmission and collided transmission, respectively. Assume that there are n vehicles competing for transmission in the network; then $p_{tra}$ can be computed by

(26) $$p_{tra}=1-{\left(1-\tau_{tra}\right)}^{n}$$

Then, according to (26), $p_{suc}$ can be calculated by

(27) $$p_{suc}^{}=\frac{p_{s}^{\prime }}{p_{tra}}=\frac{\sum_{k=1}^{n}\left[n!/(k!(n-k)!)\right]{\left(\tau_{tra}\right)}^{k}{\left(1-\tau_{tra}\right)}^{n-k}p_{cap}(i,z)}{1-{\left(1-\tau_{tra}\right)}^{n}}$$

For the basic mode, the average durations of successful transmission and failed transmission are, respectively, computed as

(28) $$\left\{\begin{array}{c} T_{s}^{bas}=T_{H}+T_{L_{p}}+T_{SIFS}+T_{ACK}+T_{DIFS}+2T_{PD}\\ T_{c}^{bas}=T_{H}+T_{L_{p}}+T_{DIFS}+T_{PD} \end{array}\right.$$

where $T_{H}$ is the transmission duration of the packet header including PHY header (${PHY}_{hdr}$) and MAC header (${MAC}_{hdr}$). $T_{L_{p}}$, $T_{SIFS}$, $T_{DIFS}$, $T_{ACK}$ and $T_{PD}$ are the durations of a successful transmission of the packet payload, SIFS, DIFS, a successful transmission of ACK and propagation delay, respectively.

For the RTS/CTS mode, the average durations of successful transmission and failed transmission can be computed as

(29) $$\left\{\begin{array}{c} T_{s}^{rts}=T_{RTS}+T_{CTS}+T_{H}+T_{L_{p}}+T_{ACK}+T_{DIFS}+3T_{SIFS}+4T_{PD}\\ T_{c}^{rts}=T_{RTS}+T_{DIFS}+T_{PD} \end{array}\right.$$

where $T_{RTS}$ and $T_{CTS}$ are the durations of successful transmissions of RTS and CTS, respectively. Besides this, the other parameters are defined the same as those in Equation (28).

### 3.4. Calculation of Average Packet Delay

The average packet delay for successfully transmitting a packet is defined as the average time from the start when the packet enters the MAC buffer queue to the end when it is successfully received. Since the *M*/*M*/1/*K* queue system is considered, if a packet from the upper layer is not discarded, it will enter the MAC buffer queue and wait to be transmitted (or be discarded by reaching the retry limit). As a result, it includes two parts, i.e., queue delay ($D_{que}$) and MAC delay ($D_{MAC}$). The former is the duration from the moment that this packet enters the MAC queue to the moment it becomes the head of the queue, and the latter is the duration from the moment it becomes the head of the queue to the moment it is successfully received. Therefore, the average packet delay can be calculated as

(30) $$D_{ave}=D_{que}+D_{MAC}$$

According to the state transition diagram for an *M/M/1/K* queue shown in Figure 3, we have

(31) $$p_{k+1}=\rho p_{k},0\leq k<K$$

where service intensity ρ can be calculated by

(32) $$\rho =\frac{\lambda_{pkt}}{\mu_{eff}}=\frac{\lambda_{pkt}}{\mu_{suc}+\mu_{dis}}$$

Then, based on Equation (31), we can obtain

(33) $$p_{k}=\rho^{k}p_{0},0\leq k\leq K$$

Subsequently, according to the normalization condition, i.e., $\sum_{j=0}^{K}p_{j}=1$, the probability that the queue of any vehicle is empty is given by

(34) $$p_{0}=\left\{\begin{array}{c} \frac{1-\rho }{1-\rho^{K+1}},\rho \neq 1\\ \frac{1}{K+1},\rho =1 \end{array}\right.$$

Therefore, combining Equations (33) and (34), the overflow probability of the MAC buffer queue can be expressed as

(35) $$p_{of}=\left\{\begin{array}{c} \frac{\rho^{K}(1-\rho )}{1-\rho^{K+1}},\rho \neq 1\\ \frac{1}{K+1},\rho =1 \end{array}\right.$$

In fact, for an *M*/*M*/1/*K* queue system, the average number of packets in the queue can be calculated as

(36) $$L_{ave}=\sum_{k=0}^{K}kp_{k}=\frac{1-\rho }{1-\rho^{K+1}}\sum_{k=1}^{K}kp_{k}=\frac{\rho \left[1-(K+1)\rho^{K}+K\rho^{K+1}\right]}{\left(1-\rho )(1-\rho^{K+1}\right)},\rho \neq 1$$

where ρ can be given by (32) and K the given maximum of queue length. It is worth pointing out that $L_{ave}=\sum_{k=0}^{K}kp_{k}=\frac{1}{K+1}\sum_{k=1}^{K}k=\frac{K}{2}$ when ρ=1. According to the Little’s formula [49], the average waiting time for a packet in the buffer queue (i.e., queue delay) can be calculated by

(37) $$D_{que}=\frac{L_{ave}}{\lambda_{pkt}(1-p_{of})}$$

where $p_{of}$ and $L_{ave}$ can be obtained by Equations (35) and (36).

For the calculation of MAC delay, it can be expressed as

(38) $$D_{MAC}=\sum_{j=0}^{M+f}E[T^{\left(j\right)}]\cdot P^{\left(j\right)}=\sum_{j=0}^{M+f}\left(T_{s}+jT_{c}+\sigma_{ave}\sum_{i=0}^{j}\frac{W_{i}-1}{2}\right)\cdot \frac{{\left(p_{c}\right)}^{j}(1-p_{c})}{1-{\left(p_{c}\right)}^{M+f+1}}$$

where $E[T^{\left(j\right)}]$ denotes the average delay of successfully transmitting a packet at backoff stage j and $P^{\left(j\right)}$ denotes the probability of the packet being successfully transmitted at backoff stage j under the premise of not being discarded. $p_{c}$ and $\sigma_{ave}$ can be obtained by Equations (15) and (25), respectively. Moreover, $W_{i}$ can be obtained by Equation (1) and $T_{s}$ and $T_{c}$ can be calculated by Equation (28) or (29).

At last, after substituting Equations (37) and (38) into Equation (30), the average packet delay can be expressed as

(39) $$D_{ave}=\frac{L_{ave}}{\lambda_{pkt}(1-p_{of})}+\sum_{j=0}^{M+f}\left(T_{s}+jT_{c}+\sigma_{ave}\sum_{i=0}^{j}\frac{W_{i}-1}{2}\right)\cdot \frac{{\left(p_{c}\right)}^{j}(1-p_{c})}{1-{\left(p_{c}\right)}^{M+f+1}}$$

where $p_{c}$, $\sigma_{ave}$, $T_{s}$, $T_{c}$, $p_{of}$, and $L_{ave}$ can be calculated by Equations (15), (25), (28) (or (29)), (35) and (36), respectively.

## 4. Model Valuation and Performance Evaluation

**Figure 4:**
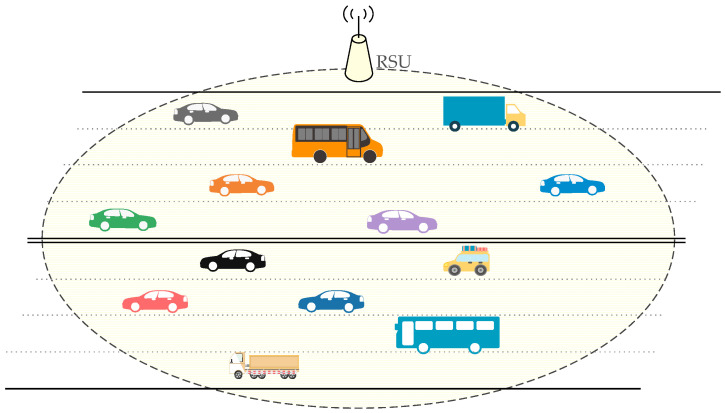
V2I communication scenario.

To validate the effectiveness of the proposed analytical model, the simulation results are given. For simplicity, the simulation scenario is that all vehicles, which are in the one-hop range of each other, communicate with an RSU, e.g., the V2I communication scenario as shown in Figure 4, like that in [54].

To verify the accuracy of the proposed model, it is compared with Zheng’s model [15] and Malone’s model [18] with one single AC queue for fairness. It is worth pointing out that Zheng’s model is still adopted in their latest work in [55]. Since the transmission rates within the range of 3 and 27 Mbps are supported by IEEE 802.11p [29,50], a 3 Mbps transmission rate is chosen in the simulation. Like in [43], the capture threshold is set to $z_{0}=2$, because a smaller value of capture threshold means that the capture effect is much more likely to come up. Moreover, the packet arrival of each buffer, which is a Possion process, is set to λ=10 pkts/s, because the performance analysis of the DCF is under the hypothesis of an unsaturated condition. The main parameters used are listed in Table 3.

### 4.1. Transmission Probability and Collision Probability

**Figure 5:**
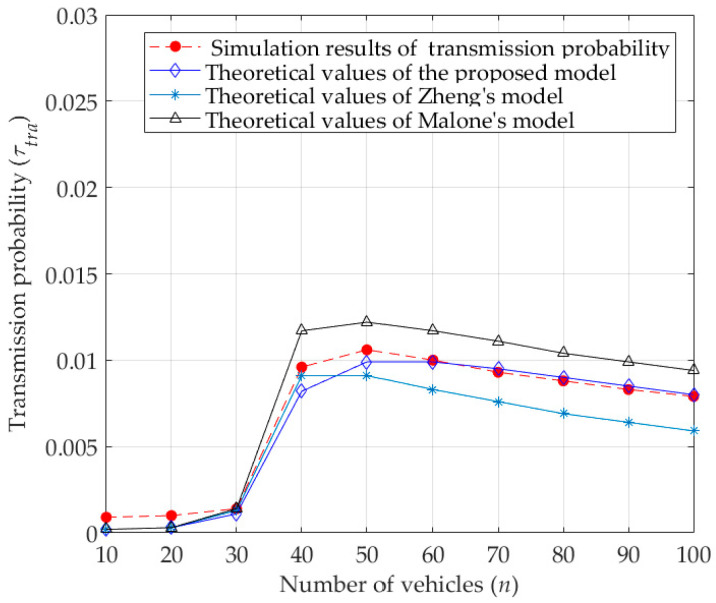
Transmission probability.

**Figure 6:**
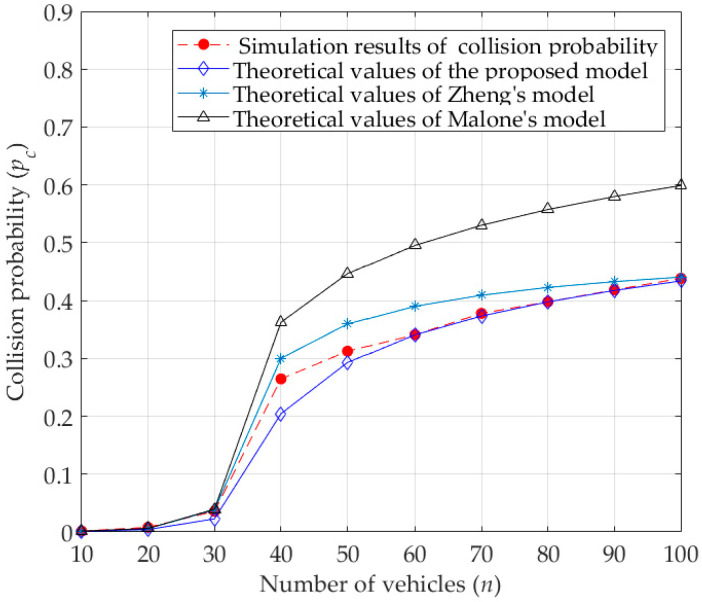
Collision probability.

According to Equations (12) and (15), the transmission probability of the vehicle ($\tau_{tra}$) is related to the minimum contention window ($W_{0}$), maximum backoff stage (M), retransmission times in the maximum backoff stage (f), the number of vehicles (n), and the probability of a collided transmission under an unsaturated condition ($p_{c}$). As shown in Figure 5 and Figure 6, with the increase of the number of vehicles, the transmission probability first increases, and then gradually decreases, while the probability of a collided transmission becomes larger and larger. Obviously, this is determined by the characteristics of the DCF protocol.

Under unsaturated conditions, when the number of vehicles is small, the possibility of a collided transmission is also small. Then, the probability of a successful transmission for packets in the buffer queue of vehicles is high, which also means that there are fewer packets waiting to be sent in the buffer queue, or even no packets waiting to be sent sometimes, resulting in a smaller transmission probability. With the increase in the number of vehicles in the network, the possibility of collided transmission increases. As a result, the vehicles need more time to successfully transmit packets, and the number of packets waiting to be transmitted in the buffer queue increases, resulting in the increase in the probability of vehicles transmitting. However, when the number of vehicles increases to a certain value, the transmission probability begins to decrease. The reason is that a high probability of collided transmissions leads to the increased possibility of delayed transmission of vehicles, which results in more time for vehicles to transmit packets, that is, the transmission probability begins to become smaller. As seen from Figure 5, compared with the theoretical results of transmission probabilities calculated by Zheng’s model and Malone’s model, the theoretical values calculated by the proposed model are much closer to the simulation results. The reason is that Malone’s model does not consider the backoff freezing mechanism, which leads to a decrease in the waiting time before transmitting packets and then an increase in the collision probability. Besides, Zheng’s model ignores the influence of capture effect, that is, the capture effect increases the transmission success rate and reduces the waiting time before transmitting packets, and then increases the transmission probability. Therefore, the transmission probabilities obtained by the proposed model, with consideration of the backoff freezing mechanism and the capture effect, are much closer to the actual transmission probabilities.

Similarly, as shown in Figure 6, since the proposed model takes the influence of the capture effect into account, one vehicle may successfully transmit among the collided vehicles. That is to say, the possibility of a collided transmission decreases. As a result, the theoretical probabilities of a collided transmission obtained by the proposed model are closer to the simulation results than that of the other two models. Therefore, when analyzing the performance of DCF (or EDCA), we should fully consider the characteristics of the protocol itself, and consider the influence of the capture effect on the protocol performance in the real IoV environment.

### 4.2. Normalized Throughput

**Figure 7:**
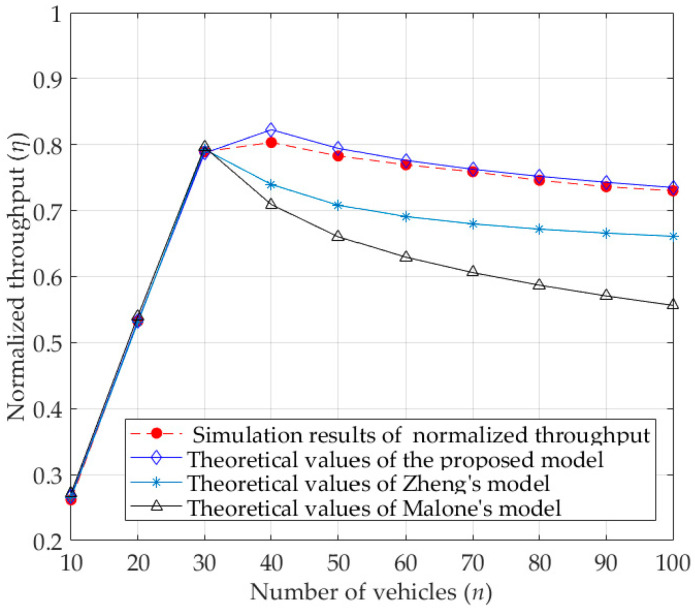
Normalized unsaturated throughput for basic access mode.

As shown in Figure 7, the values of normalized unsaturated throughput for the basic access mode under different numbers of vehicles are given. The theoretical values of normalized throughput for all models are calculated by Equation (24). As seen from Figure 7, the normalized throughput first increases with the increase in the number of vehicles, and then decreases with the increase in the number of vehicles after reaching the maximum value of normalized throughput. This is because the number of vehicles competing for channel resources is small at the beginning, and the normalized throughput gradually increases. As more and more vehicles compete for channel resources, the collision intensifies and the channel resource waste becomes more and more serious, which ultimately leads to the decrease in normalized throughput. In fact, the theoretical values of Zheng’s model are much closer to the simulation results than that of Malone’s model, because the former considers the backoff freezing mechanism. However, the theoretical values obtained by the proposed analytical model are much closer to the simulation results, and significantly higher than the theoretical values of other models when the number of vehicles is large. The reason is that the proposed analytical model not only fully considers the characteristics of the DCF protocol itself, but also considers the influence of the capture effect, thus improving the accuracy of the theoretical analysis.

**Figure 8:**
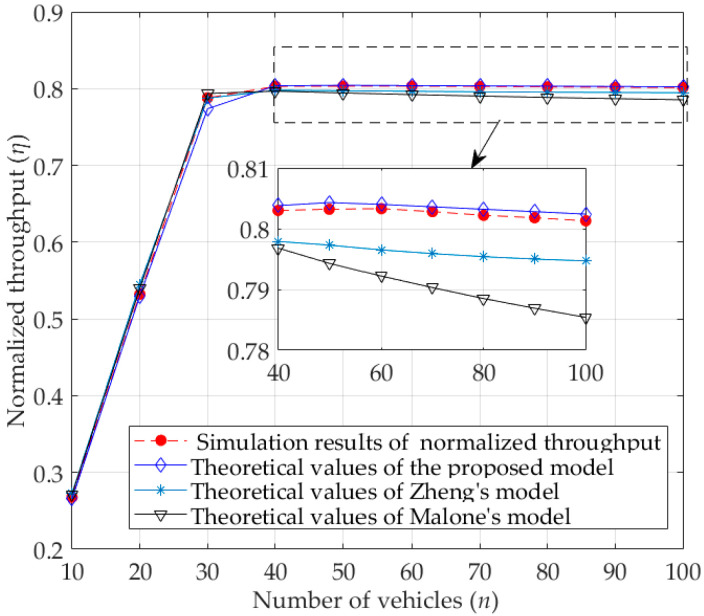
Normalized unsaturated throughput for RTS/CTS mode.

As shown in Figure 8, the values of normalized unsaturated throughput for the RTS/CTS mode first gradually increases and then slowly decreases. In addition, the normalized throughput for the RTS/CTS mode is larger than that for the basic access mode. The reason is that the RTS/CTS mechanism limits the collision to smaller control frames (i.e., RTS and CTS), effectively avoiding the collision of larger packets, thus avoiding the sharp decline in the normalized throughput as the number of vehicles increases. Moreover, when the number of vehicles is large, the theoretical values of normalized throughput obtained by the proposed model are also slightly higher than that of the other models, and much closer to the simulation results. The reason is the same as that of basic access mode. In addition, according to Figure 7 and Figure 8, it can be found that the normalized throughput for the RTS/CTS mode is less affected by the channel than for the basic mode, and with the increase in the number of vehicles, the normalized throughput of the former is significantly greater than that of the latter.

### 4.3. Average Packet Delay

**Figure 9:**
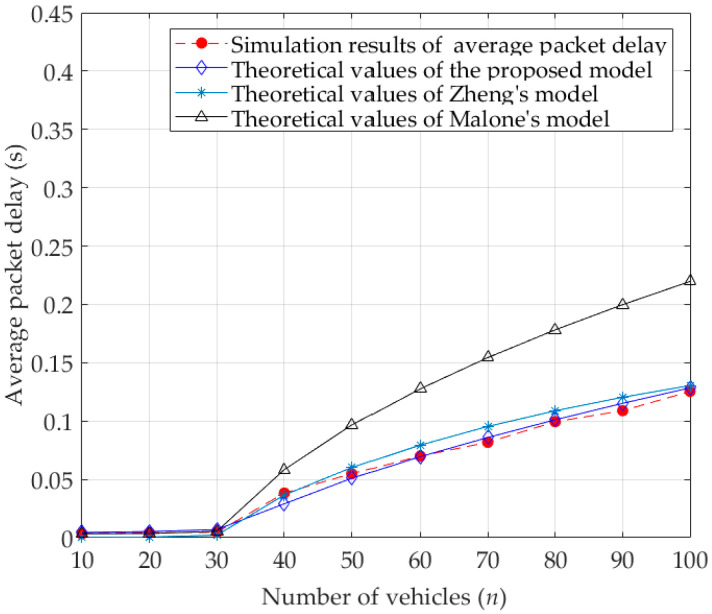
Average packet delay for basic access mode.

**Figure 10:**
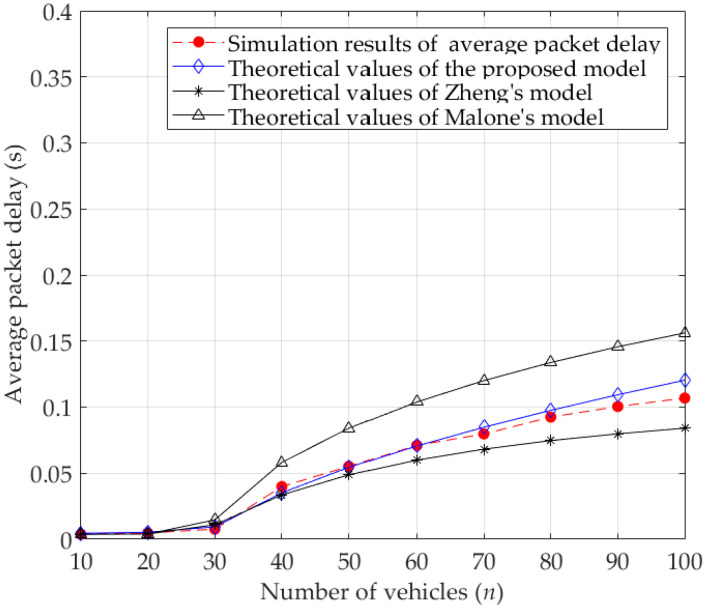
Average packet delay for RTS/CTS mode.

Figure 9 and Figure 10 give the comparisons between the theoretical values of average packet delay calculated by different analytical models and simulation results for the basic access mode and RTS/CTS mode, respectively. Among them, the theoretical values of the proposed model are calculated by Equation (37), while the theoretical values of Zheng’s model and Malone’s model are calculated by the calculation methods in the corresponding literatures [15,18], respectively. Due to the full consideration of the characteristics of the DCF protocol and the influence of capture effect, the theoretical values of average packet delay calculated by the proposed model are much closer to the simulation results.

In Figure 9, when the number of vehicles increases in the network, more vehicles competing for the wireless channel leads to intensified collision. It makes the vehicles wait for longer to transmit packets successfully, which eventually results in an increase in the average packet delay. Since the backoff freezing mechanism is ignored in Malone’s model, the collision probability calculated by this model increases, which means that the possibility of collided transmissions is amplified. Therefore, one vehicle needs more time to successfully transmit a packet, resulting in a larger average packet delay. Since both Zheng’s model and the proposed model take the backoff freezing mechanism into account, the theoretical values of average packet delay calculated by these two models are smaller than that of Malone’s model. However, compared with Zheng’s model, the theoretical values obtained by the proposed model are much closer to the simulation results, because the proposed model not only considers the influence of capture effect, but also the queuing delay.

Similarly, as shown in Figure 10, in the RTS/CTS mode, the average packet delay gradually increases along with the increase in the number of vehicles. Moreover, the theoretical values of the proposed model are much closer to the simulation results than that of the other two models, which further shows the accuracy of the proposed model. In addition, combined with Figure 9 and Figure 10, it can be found that the average packet delay for the RTS/CTS mode is lower than that for the basic access mode under the same simulation parameters. This is because the RTS/CTS mechanism limits collisions to smaller control frames (i.e., RTS and CTS), effectively avoiding collisions between larger packets.

## 5. Conclusions

In this paper, a novel analytical model of IEEE 802.11p/bd DCF with consideration of the capture effect under a Nakagami-*m* fading channel is proposed, which is more accurate than the existing analytical models and better suited to the IoV scenario. All the important characteristics of the DCF protocol and the capture effect under the Nakagami-*m* fading channel are considered in the proposed model. The accuracy of the proposed model is verified by comparison between the simulations and the analytical results, which show that the proposed model is more accurate than the existing ones, and the normalized unsaturated throughput with consideration of the capture effect is higher than that without a consideration of the capture effect. In addition, the average packet delay decreases, benefiting from the capture effect. As a result, when analyzing the DCF protocol in different communication scenarios or designing improved MAC protocols based on the DCF (or EDCA), the capture effect must be considered to make the MAC protocols more effective in real IoV scenarios. Furthermore, since the EDCA is based on the DCF with different ACs, the proposed analytical model can be easily extended to the performance analysis of the EDCA in IoV, which will be discussed in our future work.

## Figures and Tables

**Table 1:** List of abbreviations.

Abbreviation	Definition
2-D	Two-dimensional
3-D	Three-dimensional
AC	Access category
ACK	Acknowledgement
BEB	Binary exponential backoff
BER	Bit error ratio
BS	Base station
CSMA/CA	Carrier sense multiple access with collision avoidance
DCF	Distributed coordination function
DIFS	Distributed inter frame space
EDCA	Enhanced distributed channel access
EDCAF	Enhanced distributed channel access function
FIFO	Fist-in-first-out
GW	Gateway
HS	Hot spot
IoV	Internet of vehicles
MAC	Medium access control
NAV	Network allocation vector
PHY	Physical
RSU	Road-side unit
RTS/CTS	Request-to-send/clear-to-send
SIFS	Short inter-frame space
TXOP	Transmission opportunity
VANET	Vehicular ad hoc network
V2I	Vehicle-to-infrastructure
V2N	Vehicle-to-network
V2P	Vehicle-to-pedestrian
V2V	Vehicle-to-vehicle

**Table 2:** Notions used in the proposed analytical model.

Notion	Definition
$W_{j}$	Contention window of backoff stage *j*
${CW}_{min}$	Minimum contention window
${CW}_{max}$	Maximum contention window
M	Maximum backoff stage
f	Retransmission times in the maximum backoff stage
σ	Duration of a backoff slot
$\lambda_{pkt}$	Packet arrival rate of upper layer
$\lambda_{eff}$	Effective packet arrival rate
q	The probability that the cache queue is not empty
$a_{b}$	The probability of packet arrival when the channel is busy
$a_{i}$	The probability of a packet arriving when the channel is idle
$p_{i}$	The probability that the channel is idle during one backoff slot
$\tau_{tra}$	Transmission probability under unsaturated condition
$\tau_{tra}^{sat}$	Transmission probability under saturated condition
$p_{tra}$	The probability that at least one vehicle transmits
$p_{s}$	The probability of successful transmission under unsaturated condition
$p_{c}$	The probability of collided transmission under unsaturated condition
$p_{c}^{sat}$	The probability of collided transmission under saturated condition
$p_{s}^{\prime }$	The probability of successful transmission when at least one vehicle transmits
$z_{th}$	Capture threshold
m	The parameter of Nakagami fading
$p_{cap}$	The probability of capture effect
$\sigma_{ave}$	Duration of the virtual slot under unsaturated condition
$T_{b}$	Duration of busy channel
$T_{s}$	Average time for successful transmission
$T_{c}$	Average time for collided transmission
$\mu_{eff}$	Effective service rate of packets
$\mu_{suc}$	Service rate of successfully transmitted packets
$\mu_{dis}$	Overflow rate of packets
ρ	Service intensity
$p_{k}$	Steady-state probability when the queue length is *k*
$L_{p}$	The length of payload
$T_{s}^{L_{p}}$	The time required to successfully transmit the payload
$R_{t}$	The data rate
$R_{b}$	Basic transmission rate
$p_{of}$	Overflow probability of cache queue
$D_{que}$	Queue delay
$D_{MAC}$	Delay of MAC layer
$L_{ave}$	Average number of packets in the cache queue
$D_{ave}$	Average packet delay
n	Number of vehicles

**Table 3:** Simulation parameters.

Parameters	Setting
$L_{p}$	1024 bytes
${MAC}_{hdr}$	224 bits
${PHY}_{hdr}$	192 bits
*ACK*	304 bits
*RTS*	352 bits
*CTS*	304 bits
$T_{SIFS}$	32 μs
$T_{DIFS}$	58 μs
σ	13 μs
$T_{PD}$	2 μs
$R_{t}$	3 Mbps
m	1.5
$z_{th}$	2
$W_{0}$	32
$W_{M}$	1024
M	5
f	2
$\lambda_{pkt}$	10 pkts/s
Simulation time	200 s

## Data Availability

Data are contained within the article.
